# Revelando Cardiomiopatia Arritmogênica do Ventrículo Direito na Esclerodermia

**DOI:** 10.36660/abc.20230815

**Published:** 2024-07-31

**Authors:** Mehmet Rasih Sonsöz, Uğur Ozan Demirhan, Cemal Bes

**Affiliations:** 1 Departamento de Cardiologia Başakşehir Çam & Sakura City Hospital Istambul Turquia Departamento de Cardiologia – Başakşehir Çam & Sakura City Hospital, Istambul – Turquia; 2 Departamento de Reumatologia Başakşehir Çam & Sakura City Hospital Istambul Turquia Departamento de Reumatologia, Başakşehir Çam & Sakura City Hospital, Istambul – Turquia

**Keywords:** Displasia Arritmogênica Ventricular Direita, Ecocardiografia, Hipertensão Pulmonar, Escleroderma Sistêmico, Taquicardia Ventricular

## Introdução

Pacientes com esclerose sistêmica (ES) comumente relatam dispneia aos esforços e hipertensão pulmonar deve ser suspeitada primeiro, pois a prevalência nesses pacientes é relatada como sendo de 5-19% e está associada a piores desfechos clínicos.^1^ O diagnóstico diferencial adicional inclui *shunt* congênito da esquerda para a direita e cardiomiopatia arritmogênica do ventrículo direito (CAVD) quando estão presentes dilatação ventricular direita e disfunção sistólica. Nosso caso trata de uma senhora idosa com ES que foi inicialmente diagnosticada com hipertensão pulmonar pré-capilar.

## Relato de Caso

**Figura 1:**
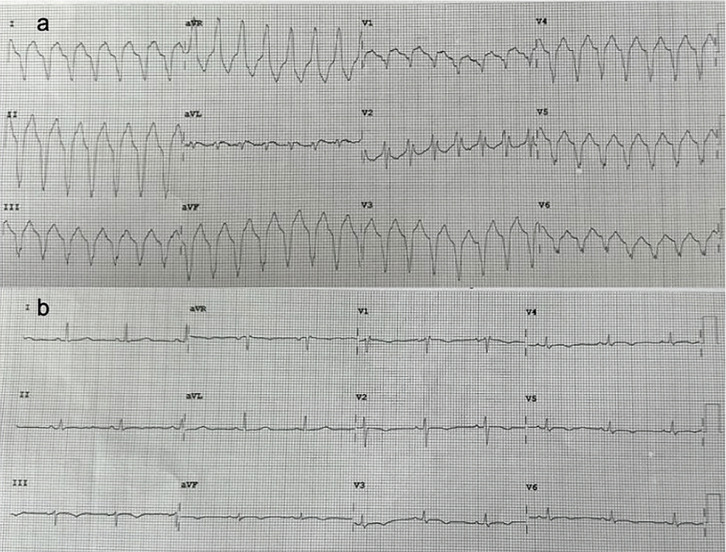
– A) Eletrocardiograma mostrando episódio de taquicardia ventricular na forma de bloqueio de ramo esquerdo. B) Eletrocardiograma demonstrando ritmo sinusal após cardioversão elétrica. Observe o bloqueio incompleto do ramo direito com ondas T negativas nas derivações II, III, aVF e V1-V6.

**Figura 2:**
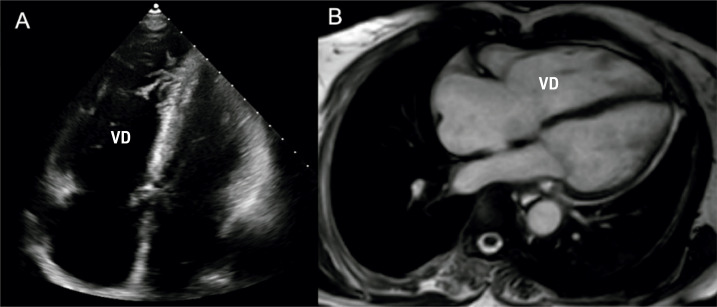
– A) Corte apical quatro câmaras ao ecocardiograma transtorácico mostrou dilatação do coração direito e redução da função sistólica do ventrículo direito. B) A ressonância magnética cardíaca revelou dilatação do coração direito, movimento dissíncrono do ventrículo direito e disfunção sistólica do ventrículo direito (fração de ejeção do VD 30%). VD: ventrículo direito.

Uma senhora de 66 anos deu entrada no ambulatório de cardiologia com dispneia crônica aos esforços. Seu histórico médico incluía ES, diagnosticada há 2 anos com base no fenômeno de Raynaud, esclerodactilia e anticorpos séricos anti-Scl70 positivos. Ela estava estável com prednisolona oral e diltiazem. Após vários meses, ela foi hospitalizada por pneumonia por COVID-19 e desenvolveu um episódio de taquicardia ventricular (TV) sustentada na forma de bloqueio de ramo esquerdo (BRE) com configuração de eixo superior. Após início da amiodarona intravenosa, o ritmo sinusal foi restaurado. O eletrocardiograma mostrou bloqueio incompleto de ramo direito com ondas T negativas nas derivações II, III, aVF e V1-V6 (Figura 1A, B). Foi encaminhada para triagem de hipertensão pulmonar (HP) e etiologia da TV. O exame cardiovascular revelou sopro sistólico no foco tricúspide e edema pré-tibial. Os exames laboratoriais revelaram um elevado peptídeo natriurético cerebral NT-pro no plasma de 789 pg/mL. O ecocardiograma transtorácico revelou função sistólica ventricular esquerda normal, dilatação das câmaras cardíacas direitas (via de saída do ventrículo direito no eixo paraesternal longo 32 mm, no eixo paraesternal curto 36 mm), função sistólica reduzida do ventrículo direito (VD) (alteração da área fracionada do VD 30% ) (Figura 2A), regurgitação tricúspide moderada (pressão sistólica da artéria pulmonar estimada em 38 mmHg). A ressonância magnética cardíaca confirmou dilatação do ventrículo direito (índice de volume diastólico final do VD: 101 mL/m^2^; índice de volume sistólico final do VD: 71mL/m^2^), movimento dissíncrono do ventrículo direito e disfunção sistólica do ventrículo direito (fração de ejeção do VD 30%) (Figura 2B). O cateterismo cardíaco revelou pressão média da artéria pulmonar discretamente elevada (21 mmHg) e resistência vascular pulmonar normal (1 unidade Wood). Portanto, a paciente foi diagnosticada com CAVD com base nos “Critérios de Pádua”.^2^ Foi planejada implantação de cardioversor-desfibrilador implantável.

## Discussão

Este caso ilustra os desafios diagnósticos que podem surgir em pacientes com ES com achados suspeitos de hipertensão pulmonar. A CAVD é uma cardiomiopatia com morfologia anormal do VD, caracterizada por AV e morte súbita cardíaca.^2^ Atualmente é considerada um subgrupo de “cardiomiopatia arritmogênica”, refletindo o conceito moderno de cardiomiopatia biventricular com envolvimento ventricular esquerdo.^2^ A coexistência de esclerodermia e CAVD, embora rara, foi relatada anteriormente.^3^ Os autores apresentaram uma paciente que foi diagnosticada com ES aos 9 anos de idade, apresentou palpitações aos 15 anos com ecocardiograma normal e desenvolveu dispneia aos esforços aos 20 anos, pois ocorreu dilatação do VD e disfunção sistólica. Os autores diagnosticaram CAVD após indução de episódio de TV na forma de BRE em um estudo eletrofisiológico e uma biópsia endomiocárdica demonstrando substituição fibrogordurosa do VD.

Outro exemplo de coexistência de duas doenças foi relatado por Arai et al.^4^ Os autores apresentaram uma mulher na faixa dos cinquenta anos com ES que desenvolveu dispneia durante o acompanhamento. O ecocardiograma mostrou disfunção sistólica e dilatação do ventrículo direito. Embora os resultados do cateterismo cardíaco direito não tenham sido compatíveis, os autores suspeitaram de hipertensão pulmonar e iniciaram bosentana. A paciente melhorou gradativamente, mas foi encontrado morta no banheiro 15 dias depois. A autópsia revelou uma parede ventricular direita fina macroscopicamente e uma substituição fibrogordurosa da parede ventricular direita histologicamente.

No nosso caso, a presença de anormalidade de repolarização no eletrocardiograma, TV na forma de BRE, dilatação ventricular direita e movimento dissíncrono sem acompanhamento de HP foram importantes pistas diagnósticas. Não realizamos biópsia endomiocárdica, pois não é necessariamente indicada para diagnóstico de CAVD, pois a ressonância magnética cardíaca foi útil para delinear tanto a função ventricular direita quanto a caracterização tecidual.

A dispneia aos esforços e os achados de insuficiência do VD em pacientes com ES devem primeiro alertar o médico para a possibilidade de HP. No entanto, a CAVD é uma cardiomiopatia rara, mas letal, e deve ser lembrada se um episódio de TV na forma de BRE for acompanhado de dilatação e disfunção sistólica do VD quando se exclui HP.
