# Supplementary material for: Revelando Cardiomiopatia Arritmogênica do Ventrículo Direito na Esclerodermia
Source: Arq Bras Cardiol. 2024 Jul 31;121(7):e20230815. [Article in Portuguese] doi: 10.36660/abc.20230815 (PMC11341208; doi:10.36660/abc.20230815)
Supplement: Translation

# Translation: Unveiling Arrhythmogenic Right Ventricular Cardiomyopathy in Scleroderma

**DOI:** 10.36660/abc.20230815i

**Authors:** Mehmet Rasih Sonsöz, Uğur Ozan Demirhan, Cemal Bes

**Affiliations:** 1 Turkey Departamento de Cardiologia – Başakşehir Çam & Sakura City Hospital, Istambul – Turkey; 2 Turkey Departamento de Reumatologia, Başakşehir Çam & Sakura City Hospital, Istambul – Turkey

**Keywords:** Arrhythmogenic Right Ventricular Dysplasia, Echocardiography, Pulmonary Hypertension, Systemic Scleroderma, Ventricular Tachycardia

## Introduction

Systemic sclerosis (SSc) patients commonly report exertional dyspnea and pulmonary hypertension should be first suspected, as the prevalence in these patients is reported to be 5-19% and it is associated with worse clinical outcomes.^1^ Additional differential diagnosis includes congenital left-to-right shunt and arrhythmogenic right ventricular cardiomyopathy (ARVC) when right ventricular dilatation and systolic dysfunction are present. Our case concerns an elderly lady with SSc who was initially diagnosed with precapillary pulmonary hypertension.

## Case Report

A 66-year-old lady presented to the outpatient cardiology clinic with chronic exertional dyspnea. Her medical history included SSc which was diagnosed 2 years ago based on Raynaud’s phenomenon, sclerodactyly, and positive serum anti-Scl70 antibodies. She was stable with oral prednisolone and diltiazem. After several months, she was hospitalized for COVID-19 pneumonia and developed an episode of sustained ventricular tachycardia (VT) in the left bundle branch block (LBBB) form with superior axis configuration. After initiation of intravenous amiodarone, sinus rhythm was restored. Her electrocardiogram showed an incomplete right bundle branch block with negative T waves in leads II, III, aVF, and V1-V6 (Figure 1a, b). She was referred for screening for pulmonary hypertension (PH) and etiology of the VT. Her cardiovascular examination revealed a systolic murmur at the tricuspid focus and pretibial edema. Laboratory tests revealed an elevated plasma NT-pro brain natriuretic peptide of 789 pg/mL. Transthoracic echocardiogram disclosed normal left ventricular systolic function, dilatation of the right heart chambers (right ventricular outflow tract in parasternal long axis 32 mm, in parasternal short axis 36 mm), reduced right ventricular (RV) systolic function (RV fractional area change 30%) (Figure 2a), moderate tricuspid regurgitation (estimated pulmonary artery systolic pressure of 38 mmHg). Cardiac magnetic resonance imaging confirmed right ventricular dilatation (RV end-diastolic volume index:101 mL/m2; RV end-systolic volume index: 71mL/m^2^), right ventricular dyssynchronous movement, and right ventricular systolic dysfunction (RV ejection fraction 30%) (Figure 2b). Cardiac catheterization disclosed mildly elevated pulmonary artery mean pressure (21 mmHg) and normal pulmonary vascular resistance (1 Wood unit). Therefore, the patient was diagnosed with ARVC based on the “Padua Criteria”.^2^ Implantation of an implantable cardioverter-defibrillator was planned.

**Figure 1:** – A) Electrocardiogram showing a ventricular tachycardia episode in the form of left bundle branch block. B) Electrocardiogram showing sinus rhythm after electrical cardioversion. Note the incomplete right bundle branch block with negative T waves in leads II, III, aVF, and V1-V6.

**Figure 2:** – A) Apical four-chamber view on transthoracic echocardiogram showed right heart dilatation and reduced right ventricular systolic function. B) Cardiac magnetic resonance imaging disclosed the right heart dilatation, right ventricular dyssynchronous movement, and right ventricular systolic dysfunction (RV ejection fraction 30%). RV: right ventricle.

## Discussion

This case illustrates the diagnostic challenges that can arise in SSc patients with suspicious findings of pulmonary hypertension. ARVC is a cardiomyopathy with abnormal RV morphology, characterized by VA and sudden cardiac death.^2^ It is now regarded as a subgroup of “arrhythmogenic cardiomyopathy,” reflecting the modern concept of biventricular cardiomyopathy with left ventricular involvement.^2^ The co-existence of scleroderma and ARVC, although rare, was reported before.^3^ The authors presented a patient who was diagnosed with SSc at 9 years of age, had palpitations at the age of 15 with normal echocardiogram findings, and developed exertional dyspnea at the age of 20, as the RV dilatation and systolic dysfunction occurred. The authors diagnosed ARVC after inducing a VT episode in LBBB form on an electrophysiologic study and an endomyocardial biopsy demonstrating fibrofatty replacement of the RV.

Another example of the coexistence of two diseases was reported by Arai et al.^4^ The authors presented a woman in her fifties with SSc who developed dyspnea during follow-up. The echocardiogram showed right ventricular systolic dysfunction and dilatation. Although the results of right heart catheterization were not compatible, the authors suspected pulmonary hypertension and started her on bosentan. The patient gradually improved but was found dead in the bathroom 15 days later. The autopsy revealed a thin right ventricular wall macroscopically and fibrofatty replacement of the right ventricular wall histologically.

In our case, the presence of the repolarization abnormality on the electrocardiogram, VT in LBBB form, right ventricular dilatation, and dyssynchronous movement without accompanying PH were major diagnostic clues. We did not perform an endomyocardial biopsy, as it is not necessarily indicated to diagnose ARVC, because cardiac magnetic resonance imaging was useful for delineating both the right ventricular function and tissue characterization.

The exertional dyspnea and findings of RV failure in SSc patients should first alert the clinician to the possibility of PH. However, ARVC is a rare but lethal cardiomyopathy and should be kept in mind if an episode of VT in LBBB form is accompanied by dilatation and systolic dysfunction of the RV when PH is excluded.
